# Integrated Multi-omics Profiling of 2,4-dinitrochlorobenzene (DNCB)-induced Atopic Dermatitis in Mice Reveals a Coordinated Network of Barrier Dysfunction, Immune Activation, and Metabolic Reprogramming

**DOI:** 10.1007/s10753-026-02553-z

**Published:** 2026-06-27

**Authors:** Wenxiang Liu, Xu Li, Zhihui Wan, Jieshi Xie, Shu Wang, Yanhong Zhai, Zheng Cao, Ping Wang, Cheng Wang

**Affiliations:** 1 Department of Laboratory Medicine, Beijing Obstetrics and Gynecology Hospital, Capital Medical University, Beijing Maternal and Child Health Care Hospital, Beijing, 100026 China; 2 Department of Allergy, Shandong Institute of Respiratory Diseases, Shandong Provincial Qianfoshan Hospital, The First Affiliated Hospital of Shandong First Medical University, Jinan, 250013 China; 3 Institute of Traditional Chinese Medicine Pharmacology, Shandong Academy of Chinese Medicine, Jinan, 250014 Shandong China

**Keywords:** DNCB, Atopic dermatitis mouse model, Multi-omics integration, Cornified envelope, Keratinocyte

## Abstract

Atopic dermatitis (AD) is caused by a combination of epidermal barrier defect and immune imbalance. However, the molecular networks between these structural abnormalities and metabolic variations are unclear. This study aim of this research was to examine the concurrent molecular alterations in skin barrier damage and metabolic disorders in an AD-like mouse model by a multi-omics strategy. A 2,4-dinitrochlorobenzene (DNCB)-induced AD-like mouse model was established and the skin tissues were examined through the combination of transcriptomic, quantitative proteomic, and metabolomic analyses. Cross-omics correlation and network analyses were performed to identify consistently abnormal molecular pathways and crucial regulatory molecules. DNCB treatment caused severe epidermal hyperplasia, and prominent infiltration of CD3⁺ T cells, F4/80⁺ macrophages, and mast cells. Transcriptomic and proteomic analysis indicated significant disruption in keratinocyte differentiation, extracellular matrix organization, and cornified envelope formation pathways. Combined analysis detected 171 molecules which were simultaneously altered at both mRNA and protein levels, and network analysis identified FLG2 and KRT6B as central barrier-related molecules. Pathway enrichment analysis consistently showed the participation of AMPK and PPAR signaling pathways. Metabolomic analysis also revealed coordinated changes in lipid and amino acid metabolism which were closely associated with cornified envelope-associated genes and collagen-modifying enzymes. These findings indicate a close relationship between barrier, immune and metabolic regulation in DNCB-induced dermatitis and provide a multi-omics resource for future mechanistic studies of atopic skin inflammation.

## Introduction

Atopic dermatitis (AD) is a chronic relapsing inflammatory skin disease mainly driven by defects in the epidermal barrier, enhanced immune cell infiltration, and significant alterations in cutaneous immunity and metabolism [1]. As one of the most prevalent inflammatory dermatoses worldwide, AD imposes a substantial burden on quality of life and health systems [1]. Although its pathogenesis is multifactorial, involving genetic susceptibility, immune dysregulation, and environmental triggers, disruption of epidermal barrier integrity and aberrant keratinocyte differentiation are now recognized as central pathogenic events [2]. Structural proteins involved in cornified envelope formation, including filaggrin family members and keratins, play indispensable roles in maintaining stratum corneum integrity and hydration capacity [3]. Defects in these components lead to increased transepidermal water loss, altered skin surface microenvironment, and enhanced susceptibility to allergen penetration and immune activation [4].

Beyond structural abnormalities, AD is characterized by robust immune activation involving T lymphocytes, macrophages, and mast cells, accompanied by cytokine-driven signaling cascades [5, 6]. Increasing evidence suggests that these inflammatory processes are tightly coupled with metabolic reprogramming in the skin [7]. Metabolic processes including lipid metabolism and fatty acid oxidation, along with signaling pathways mediated by AMP-activated protein kinase (AMPK) and peroxisome proliferator-activated receptor (PPAR), are increasingly recognized as important regulators of epidermal differentiation, barrier lipid homeostasis, and inflammatory activity [8–10]. Perturbations in these metabolic pathways may therefore amplify barrier dysfunction and sustain chronic inflammation, highlighting the value for multi-omics approaches for capturing the complex molecular landscape of AD across different biological layers [11].

However, these structural, immune, and metabolic abnormalities do not occur independently. Increasing evidence suggests that complex inflammatory diseases such as AD involve molecular alterations across multiple biological layers, including gene expression, protein abundance, and metabolic activity [11]. Because each omics platform captures only a subset of biological information, analyses restricted to a single molecular layer may provide an incomplete view of disease-associated processes [12]. Multi-omics approaches therefore offer an opportunity to characterize complementary aspects of disease biology and to identify biological pathways that show consistent alterations across different molecular levels. Supporting this concept, studies in diverse disease settings have demonstrated that multi-omics analyses can reveal molecular relationships and disease-associated signatures that are not readily apparent from individual datasets alone [13]. This conceptual framework motivates the present study, which employs complementary transcriptomic, proteomic, and metabolomic profiling to characterize molecular alterations associated with barrier dysfunction, immune activation, and metabolic perturbation in AD-like skin inflammation.

Dermatitis induced by 2,4-dinitrochlorobenzene (DNCB) is commonly employed as an experimental model that reproduces major pathological features of AD, including epidermal hyperplasia, immune cell infiltration, and barrier impairment [14–16]. Given the multifactorial nature of AD pathogenesis, this model provides a useful experimental platform for examining transcriptomic, proteomic, and metabolomic alterations within the same biological context. However, while individual transcriptomic or proteomic alterations have been described, the coordinated regulation across multiple molecular layers including transcriptome, proteome, and metabolome remains incompletely characterized [17]. A broader characterization of how structural, immune, and metabolic alterations are reflected across transcriptomic, proteomic, and metabolomic layers may provide valuable insights into biological processes associated with DNCB-induced skin inflammation.

In the present study, we established a DNCB-induced AD-like mouse model and performed transcriptomics, quantitative proteomics, and metabolomics profiling. Through cross-platform comparisons and pathway analyses, we aimed to identify concordantly altered molecular signatures and biological pathways associated with epidermal barrier disruption and metabolic remodeling. Our findings provide a multi-omics characterization of coordinated alterations associated with epidermal barrier dysfunction, immune activation, and metabolic perturbation in DNCB-induced dermatitis. These results establish a reference resource for future mechanistic studies and hypothesis-driven experimental validation, contributing to a broader understanding of molecular alterations associated with atopic skin inflammation across complementary biological layers.

## Materials and Methods

### Animals

Six-week-old male BALB/c mice were obtained from Jinan Pengyue Experimental Animal Breeding Co., Ltd. (Jinan, China) and maintained in an SPF facility under controlled environmental conditions with unrestricted access to food and water. All animal procedures were approved by the Animal Ethics Committee of the Shandong Academy of Chinese Medicine (Approval No. SDZYY20240304004) and complied with institutional animal care guidelines.

After a 7-day acclimatization period, mice were randomly assigned to two groups (*n* = 10 per group): a vehicle control group and a DNCB-treated dermatitis group. Mice were briefly anesthetized, and the hair on the dorsal region was removed 24 h prior to the start of treatment. Mice assigned to the DNCB group underwent a sensitization phase in which a 1% DNCB solution (100 µL; Sigma-Aldrich, USA) prepared in acetone/olive oil (1:3, v/v) was applied topically on days 1, 4, and 7. To establish chronic dermatitis, 100 µL of 0.5% DNCB was subsequently administered repeatedly to the previously shaved dorsal skin together with both auricles on days 14, 17, 20, 23, 26, 29, and 32. Mice in the control group received the corresponding vehicle following the identical treatment schedule. Twenty-four hours after the final exposure (day 33), animals were anesthetized using inhaled isoflurane (2.5%; RWD Life Science) delivered with oxygen, followed by humane sacrifice via cervical dislocation. Skin specimens from the dorsal region were then excised and processed for downstream analyses.

### Skin Tissue Collection and Histological Analysis

After the final DNCB challenge, mice were euthanized, and dorsal skin tissues were collected. The tissues were preserved in 4% paraformaldehyde, subsequently processed for paraffin embedding, and cut into 5 μm sections. Histological evaluation was performed using hematoxylin and eosin staining (H&E, C0105S, Beyotime, Shanghai, China) following standard protocols. Epidermal thickness was quantitatively assessed from stained sections using ImageJ (NIH, USA).

### Immunohistochemistry (IHC)

Immunohistochemical analysis was conducted on paraffin-embedded skin sections following standard histological processing. Briefly, tissue sections were prepared through dewaxing and graded rehydration prior to antigen retrieval. Prior to antibody incubation, tissue sections were exposed to 3% hydrogen peroxide to quench endogenous peroxidase activity. Sections were incubated overnight at 4 °C with primary antibodies against CD3 (T lymphocytes, ab16669, Abcam) and F4/80 (macrophages, ab6640, Abcam). After washing, appropriate horseradish peroxidase–linked secondary antibodies were applied, and immunoreactivity was visualized using a diaminobenzidine (DAB) chromogenic system. Hematoxylin was used for nuclear counterstaining. Mast cell infiltration was evaluated by toluidine blue staining. For quantitative assessment, positively stained cells were enumerated using ImageJ software in five randomly selected high-power microscopic fields from each sample.

### RNA sequencing and transcriptomic analysis

Total RNA was isolated from frozen skin samples using TRIzol reagent (Invitrogen). For library preparation, 400 ng of total RNA was used as input with the NEBNext Ultra II RNA Library Preparation Kit. Libraries were sequenced on an Illumina NovaSeq 6000 platform with 150 bp paired-end reads. Raw reads were quality-controlled with FastQC and aligned to the mouse reference genome (GRCm38) using STAR. Differential expression analysis was performed with DESeq2 (adjusted p-value < 0.05, |log₂FC| > 1). Gene Ontology (GO) annotation and Kyoto Encyclopedia of Genes and Genomes (KEGG) pathway enrichment analyses of differentially expressed genes were performed using the clusterProfiler package in R.

### Proteomic profiling and data analysis

Following homogenization of skin tissues in lysis buffer, proteins were isolated, and 10 µg of protein per sample was subsequently subjected to reduction, alkylation, and trypsin digestion. TMT 16-plex–labeled peptides were fractionated by high-pH reverse-phase chromatography and analyzed by LC–MS/MS on a Q Exactive HF-X mass spectrometer coupled to an Easy-nLC 1200 system. Raw data were processed using Proteome Discoverer 2.5 against the UniProt mouse database. After normalization of protein abundance, differential expression analysis was conducted using a moderated t-test. Proteins with an adjusted *p* value < 0.05 and |log₂ fold change| > 1 were considered significantly altered. GO and KEGG enrichment analyses were then carried out to explore their functional relevance.

### RT-qPCR and western blot validation

To validate the transcriptomic and proteomic findings, expression of *Krt6b* was examined by RT-qPCR and Western blotting. Total RNA and protein were extracted from skin tissues using standard procedures. Relative *Krt6b* mRNA expression was quantified by SYBR Green-based RT-qPCR and normalized to *Gapdh* using the 2^^−ΔΔCt^ method. Primer sequences were as follows: *Gapdh*-F: AGGTCGGTGTGAACGGATTTG; *Gapdh*-R: TGTAGACCATGTAGTTGAGGTCA; *Krt6b*-F: CAGAACCTGGAGCCTATGTTTGA; *Krt6b*-R: TGCAGTTCAACCTTGGTCATGTA. For Western blotting, proteins were separated by SDS-PAGE, transferred to PVDF membranes, and probed with antibodies against KRT6B (17391-1-AP, Proteintech) and GAPDH (60004-1-Ig, Proteintech). Protein abundance was quantified by densitometric analysis using AlphaView SA software (ProteinSimple, USA).

### Integrated transcriptomic and proteomic analysis

Pearson’s correlation analysis was conducted to evaluate the consistency between transcriptomic and proteomic alterations. Molecules with consistent up- or down-regulation (differential expression genes or proteins) were identified. Overlapping molecules were analyzed for functional enrichment and protein-protein interactions (PPI) using the STRING database, and the networks were visualized in Cytoscape.

### Immunofluorescence (IF) Staining

The skin sections were fixed, permeabilized, and blocked with 5% BSA. Primary antibodies against KRT6B (17391-1-AP, Proteintech) and FLG2 (bs-16100R, Bioss) were applied to the prepared skin sections and allowed to bind at 4 °C overnight. After removal of unbound antibodies, secondary antibodies were applied to visualize the target proteins. Nuclear staining was performed using DAPI, and fluorescence signals were subsequently recorded using a fluorescence imaging system.

### Metabolomic profiling and pathway analysis

Metabolites were isolated from skin tissues (100 mg) using an organic solvent extraction mixture composed of methanol, acetonitrile, and water (2:2:1, v/v/v, prechilled 80% methanol protocol). The homogenate was vortexed, incubated on ice for 5 min, and centrifuged at 15,000 × g, 4℃ for 20 min. The supernatant was diluted to a final concentration containing 53% methanol by LC–MS grade water, transferred to a fresh tube, and centrifuged again at 15,000 × g, 4 °C for 20 min. The resulting extracts were subjected to metabolomic profiling by ultra-performance liquid chromatography coupled with tandem mass spectrometry (UHPLC–MS/MS) using a Vanquish UHPLC system coupled with an Orbitrap Q Exactive™ HF-X mass spectrometer (Thermo Fisher), with data acquired under both positive and negative electrospray ionization conditions (spray voltage ± 3.5 kV, capillary temperature 320 °C, sheath gas 35 arb, aux gas 10 arb). Samples were injected onto a Hypersil Gold column (100 × 2.1 mm, 1.9 μm) using a 12-min linear gradient at a flow rate of 0.2 mL/min, with eluent A (0.1% FA in water) and eluent B (methanol). Raw spectral data were processed for feature detection, retention time alignment, and signal normalization using Progenesis QI software Metabolites were identified by matching against the NovoMetDB database with a mass deviation < 10 ppm. Missing values (> 50%) were filtered, and remaining missing values were imputed using KNN. QC sample-based normalization was applied, and metabolites with CV > 30% in QC samples were excluded. Multivariate statistical analyses were subsequently carried out in the SIMCA-P environment to characterize metabolic variation between groups, including principal component analysis (PCA) and partial least squares discriminant analysis (PLS-DA). Metabolites were considered significantly altered when they exhibited a variable importance in projection (VIP) score greater than 1.0, a p value below 0.05, and an absolute log₂ fold change exceeding 1. The biological pathways associated with these metabolites were further explored using KEGG-based enrichment analysis implemented in MetaboAnalyst 5.0. Spearman’s rank correlation was applied to assess potential associations between metabolite abundance and gene expression.

### Statistical analysis

Data analysis was conducted with GraphPad Prism 8 (GraphPad Software, USA) together with R software (version 4.3.0). Group differences between control and DNCB-treated mice were assessed using an unpaired two-sided Student’s *t*-test. All bar graphs are presented as the mean ± standard deviation (SD) unless otherwise specified. A p-value < 0.05 was considered statistically significant (*p < 0.05, **p < 0.01, ***p < 0.001).

## Results

### Establishment and phenotypic analysis of a murine dermatitis model induced by DNCB

**Fig. 1:**
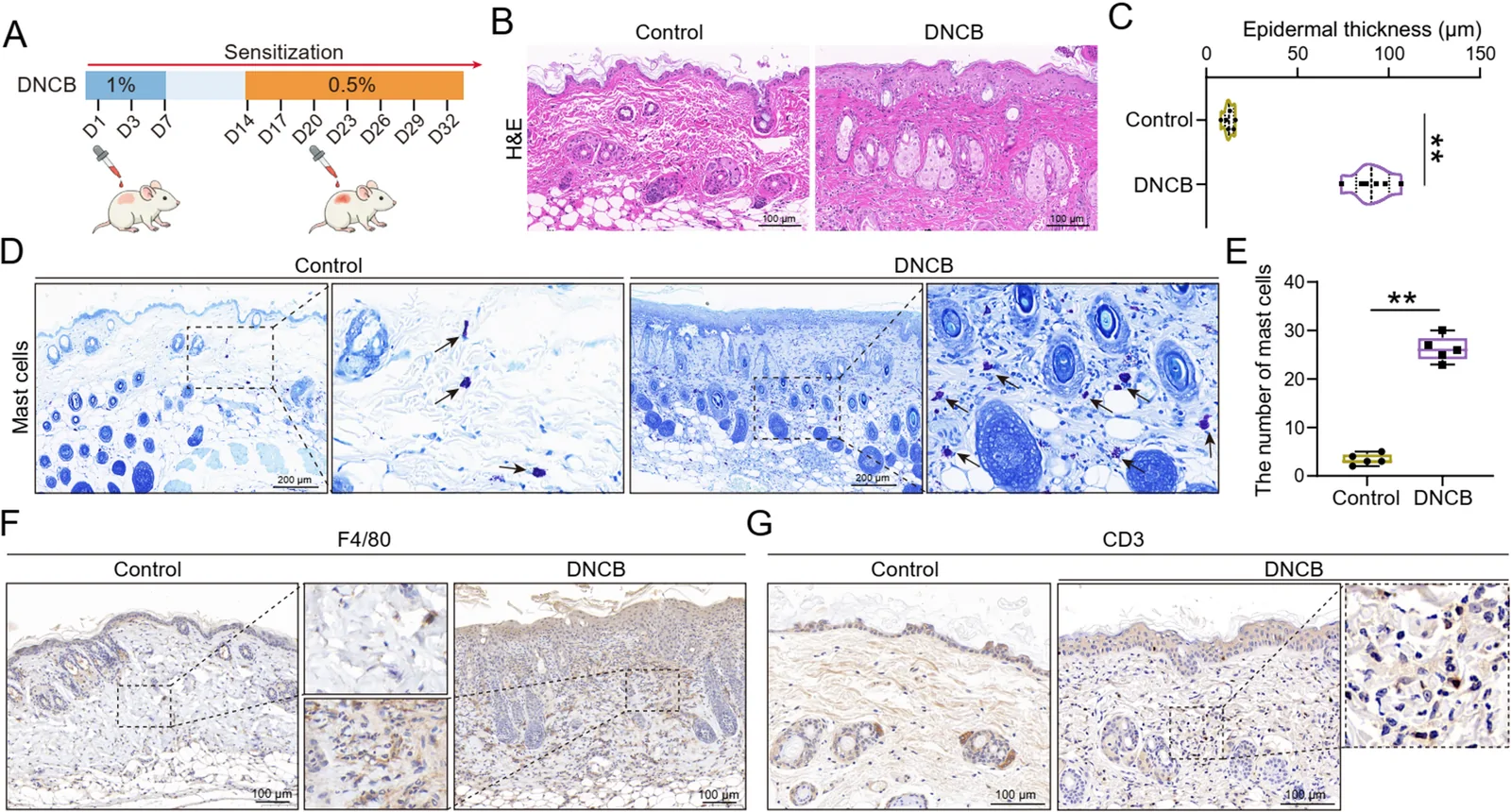
DNCB-induced dermatitis recapitulates key pathological features of atopic dermatitis in mice.**A** Schematic diagram of the experimental design for sensitization and challenge with DNCB to establish an atopic dermatitis-like murine model. **B** Representative H&E stained skin sections from control and DNCB-treated mice. Scale bar, 100 μm. **C** Quantification of epidermal thickness from H&E-stained sections. **D** Representative images of toluidine blue staining for mast cells. Scale bars, 200 μm. **E** Quantitative analysis of mast cell numbers. **F** Representative images of F4/80⁺ macrophage staining in control and DNCB-treated mice. Scale bars, 200 μm. **G** Representative images of CD3⁺ T lymphocyte staining in control and DNCB-treated mice. Scale bars, 200 μm.

As illustrated in Fig. 1A, mice were sensitized and subsequently challenged with DNCB according to the established experimental protocol to generate an AD-like model. H&E-stained skin sections from DNCB-treated mice exhibited significant epidermal hyperplasia and architectural abnormalities compared with those from control mice (Fig. 1B). Consistent with histological observations, epidermal thickness was significantly elevated in DNCB-treated mice (Fig. 1C), reflecting pronounced epidermal hyperproliferation.

Inflammatory cell infiltration was further assessed. Toluidine blue staining demonstrated a substantial increase in dermal mast cell accumulation in DNCB-treated mice relative to controls (Fig. 1D), which was supported by quantitative analysis using ImageJ software (Fig. 1E). In addition, immunostaining revealed enhanced infiltration of F4/80⁺ macrophages within DNCB-induced lesions (Fig. 1F). Similarly, CD3⁺ T lymphocytes were significantly increased in DNCB-treated mice relative to controls (Fig. 1G). These results indicate that DNCB treatment recapitulates key pathological features of human AD in mice.

### Proteomic profiling reveals extensive extracellular matrix remodeling and cornified envelope dysregulation in DNCB-treated skin

**Fig. 2:**
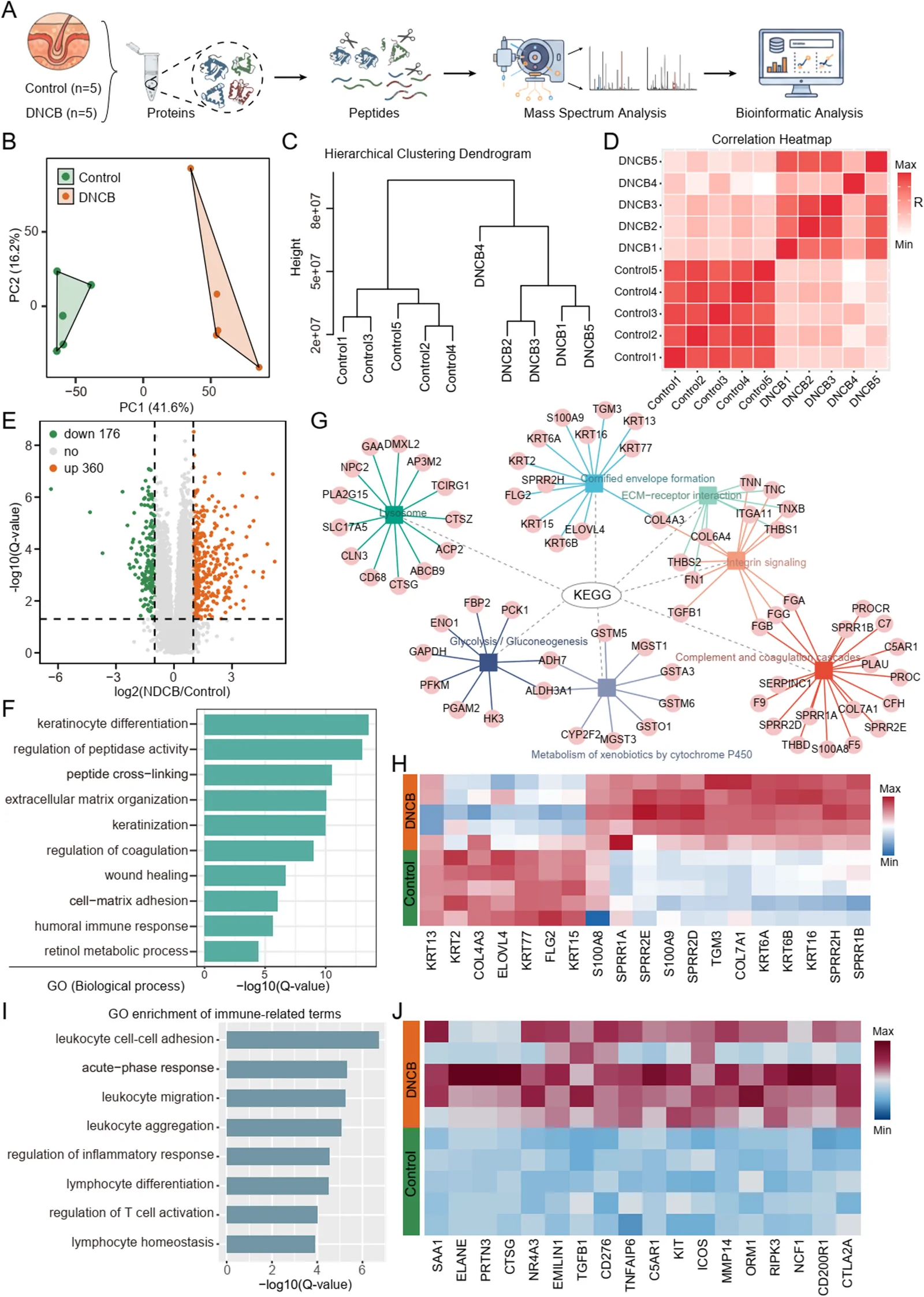
Proteomic profiling reveals extensive extracellular matrix remodeling and cornified envelope dysregulation in DNCB-treated skin. **A** Schematic workflow of the quantitative proteomic analysis performed on skin tissues from control and DNCB-treated mice (*n* = 5 per group). **B** PCA plot of the proteomic profiles, showing clear separation between the control and DNCB-treated groups. **C** Unsupervised hierarchical clustering of all quantified proteins revealed distinct proteomic patterns between the two groups. **D** Correlation analysis matrix of proteomic profiles within and between control and DNCB-treated groups. **E** Volcano plot showing differentially expressed proteins between DNCB-treated and control groups. **F** Gene Ontology (GO) biological process enrichment analysis of differentially expressed proteins. **G** Kyoto Encyclopedia of Genes and Genomes (KEGG) pathway enrichment analysis of differentially expressed proteins. **H** Heatmap showing expression levels of proteins involved in the cornified envelope formation pathway in control and DNCB-treated groups. **I** Gene Ontology enrichment of immune-related biological processes among differentially expressed proteins. **J** Expression heatmap of immune activation proteins in control versus DNCB-treated groups.

To characterize global protein alterations induced by DNCB, skin tissues from control and DNCB-treated mice (*n* = 5 per group) were subjected to quantitative proteomic analysis (Fig. 2A). PCA revealed a clear distinction between DNCB-treated and control mice, indicating distinct proteomic signatures (Fig. 2B). Consistently, unsupervised hierarchical clustering and correlation analyses further confirmed robust discrimination between groups (Fig. 2C and D).

Differential expression analysis identified 360 significantly upregulated and 176 significantly downregulated proteins in the DNCB group compared with controls (Fig. 2E), highlighting widespread proteomic reprogramming. Functional enrichment analysis of differentially expressed proteins indicated significant involvement in keratinocyte differentiation, extracellular matrix organization, peptide cross-linking, and regulation of peptidase activity (Fig. 2F), suggesting structural remodeling and altered epidermal differentiation. KEGG pathway enrichment analysis further demonstrated significant enrichment in complement and coagulation cascades, cornified envelope formation, and lysosome pathways (Fig. 2G), reflecting activation of inflammatory and barrier-related processes. Notably, key genes involved in cornified envelope formation were markedly dysregulated (Fig. 2H), indicating impaired epidermal barrier integrity and aberrant keratinocyte activation in DNCB-induced dermatitis.

Beyond the structural and epidermal processes shown in Fig. 2F, GO enrichment also identified immune-related pathways, including leukocyte cell-cell adhesion, acute-phase response, leukocyte migration, and others (Fig. 2I). These GO terms correspond directly to the immune cell types detected by histological staining and provide molecular evidence for the cellular infiltration. A focused heatmap showed coordinated upregulation of proteins linked to mast cells (KIT, CTSG), macrophages (MMP14, C5AR1, CD200R1, EMILIN1), T lymphocytes (ICOS, CD276, CTLA2A, NR4A3), neutrophils (ELANE, PRTN3, NCF1), and acute-phase or inflammatory mediators (SAA1, ORM1, TNFAIP6, TGFB1, RIPK3) in the DNCB-treated group (Fig. 2J).

### Transcriptomic profiling reveals coordinated alterations in epidermal differentiation, lipid metabolism, and inflammatory signaling in DNCB-treated skin

**Fig. 3:**
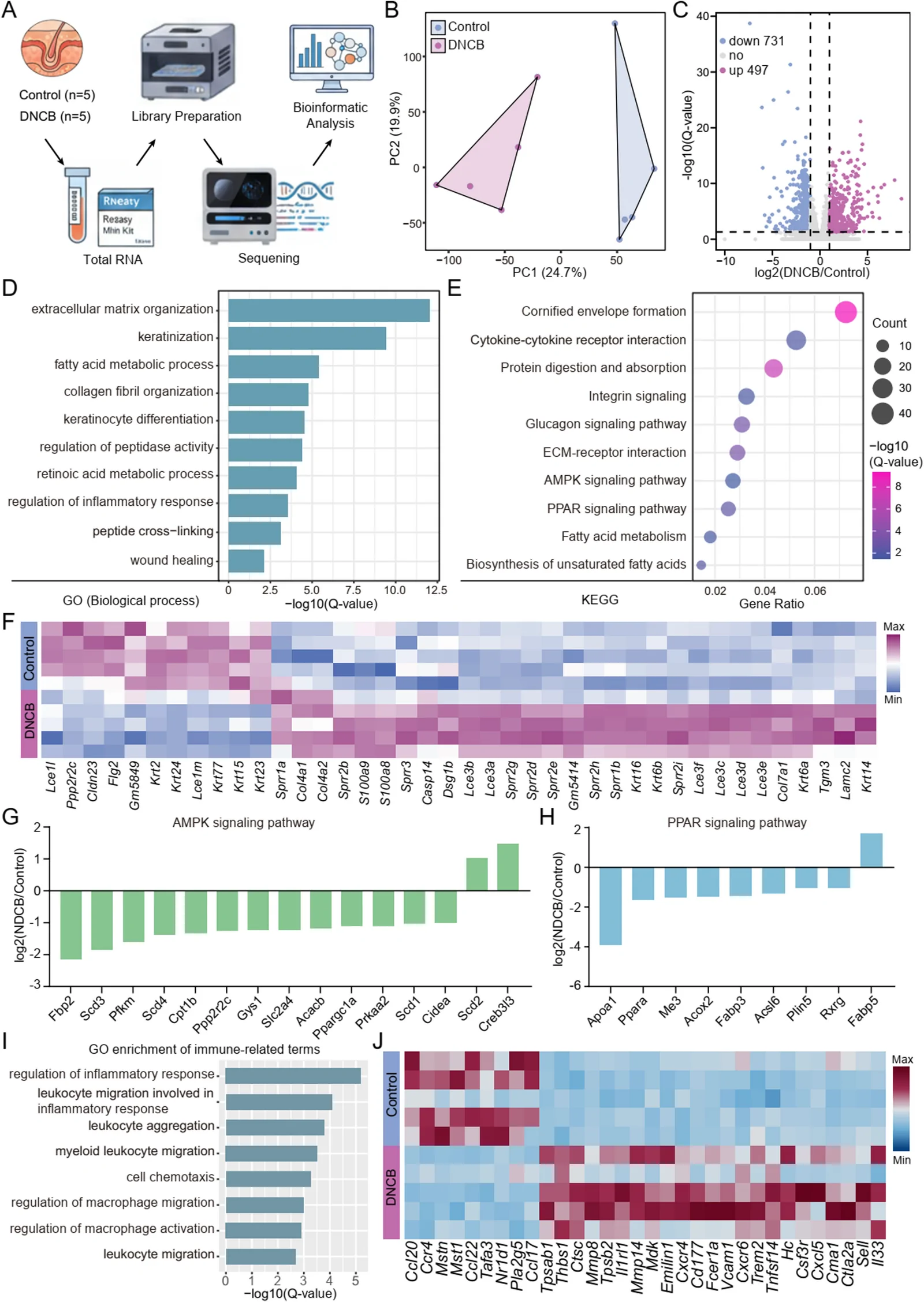
Transcriptomic analysis of DNCB-treated skin.**A** Schematic workflow of RNA sequencing performed on skin tissues from control and DNCB-treated mice. **B** PCA of transcriptomic profiles from control and DNCB-treated groups. **C** Volcano plot showing differentially expressed genes between DNCB-treated and control groups. **D** GO biological process enrichment analysis of differentially expressed genes. **E** KEGG pathway enrichment analysis of differentially expressed genes. **F** Heatmap showing expression levels of genes involved in the cornified envelope formation pathway in control and DNCB-treated groups. **G** Bar chart showing the log₂(fold change) in expression of AMPK signaling pathway genes. **H** Bar chart showing the log₂(fold change) in expression of PPAR signaling pathway genes. **I** Gene Ontology enrichment of immune-related biological processes among differentially expressed genes. **J** Expression heatmap of immune activation genes in control versus DNCB-treated groups.

To further delineate transcriptional changes induced by DNCB exposure, RNA sequencing was performed on skin tissues from control and DNCB-treated mice (*n* = 5 per group) (Fig. 3A). PCA demonstrated clear segregation between the DNCB and control groups, indicating distinct global transcriptional profiles (Fig. 3B). Differential expression analysis identified 497 upregulated and 731 downregulated genes in the DNCB group compared with controls (Fig. 3C), reflecting extensive transcriptional reprogramming. Functional enrichment analysis indicated that the differentially expressed genes were mainly involved in extracellular matrix organization, keratinization, and fatty acid metabolic process (Fig. 3D), suggesting coordinated disruption of structural integrity, epidermal differentiation, and lipid homeostasis.

KEGG pathway analysis further revealed significant enrichment in cornified envelope formation, cytokine-cytokine receptor interaction, and protein digestion and absorption pathways (Fig. 3E), consistent with activation of inflammatory responses and barrier dysfunction. Notably, a substantial number of genes within the Cornified envelope formation pathway were significantly dysregulated (Fig. 3F), in strong agreement with the proteomic findings. Importantly, multiple genes involved in the AMPK and PPAR signaling pathway, key regulators of AD development and epidermal lipid metabolism, were significantly dysregulated [9, 10], were also significantly altered (Fig. 3G and H), highlighting metabolic signaling perturbations in DNCB-induced skin inflammation.

Similar to the proteomic results, GO enrichment analysis of the differentially expressed genes also identified immune-related biological processes, including regulation of inflammatory response, leukocyte migration involved in inflammatory response, leukocyte aggregation, and others (Fig. 3I). A heatmap of immune activation genes showed a coordinated expression shift in the DNCB group relative to controls (Fig. 3J). The altered genes included those linked to mast cells (*Cma1*, *Tpsb2*, *Tpsab1*, *Fcer1a*), macrophages (*Trem2*, *Mmp14*, *Mdk*, *Emilin1*, *Thbs1*, *Mmp8*, *Ctsc*, *Hc*), T lymphocytes and chemotaxis (*Ccl20*, *Ccl22*, *Ccl17*, *Ccr4*, *Cxcr4*, *Cxcr6*, *Sell*, Ctla2a, *Vcam1*, *Tnfsf14*, *Mst1*, Mstn), neutrophils (*Cd177*, *Cxcl5*, *Csf3r*), and inflammatory mediators (*Il33*, *Il1rl1*, *Nr1d1*, *Pla2g5*, *Tafa3*), consistent with the immune cell infiltration observed at the tissue level.

### Integration of transcriptomic and proteomic analyses reveals coordinated molecular changes in DNCB-induced skin

**Fig. 4:**
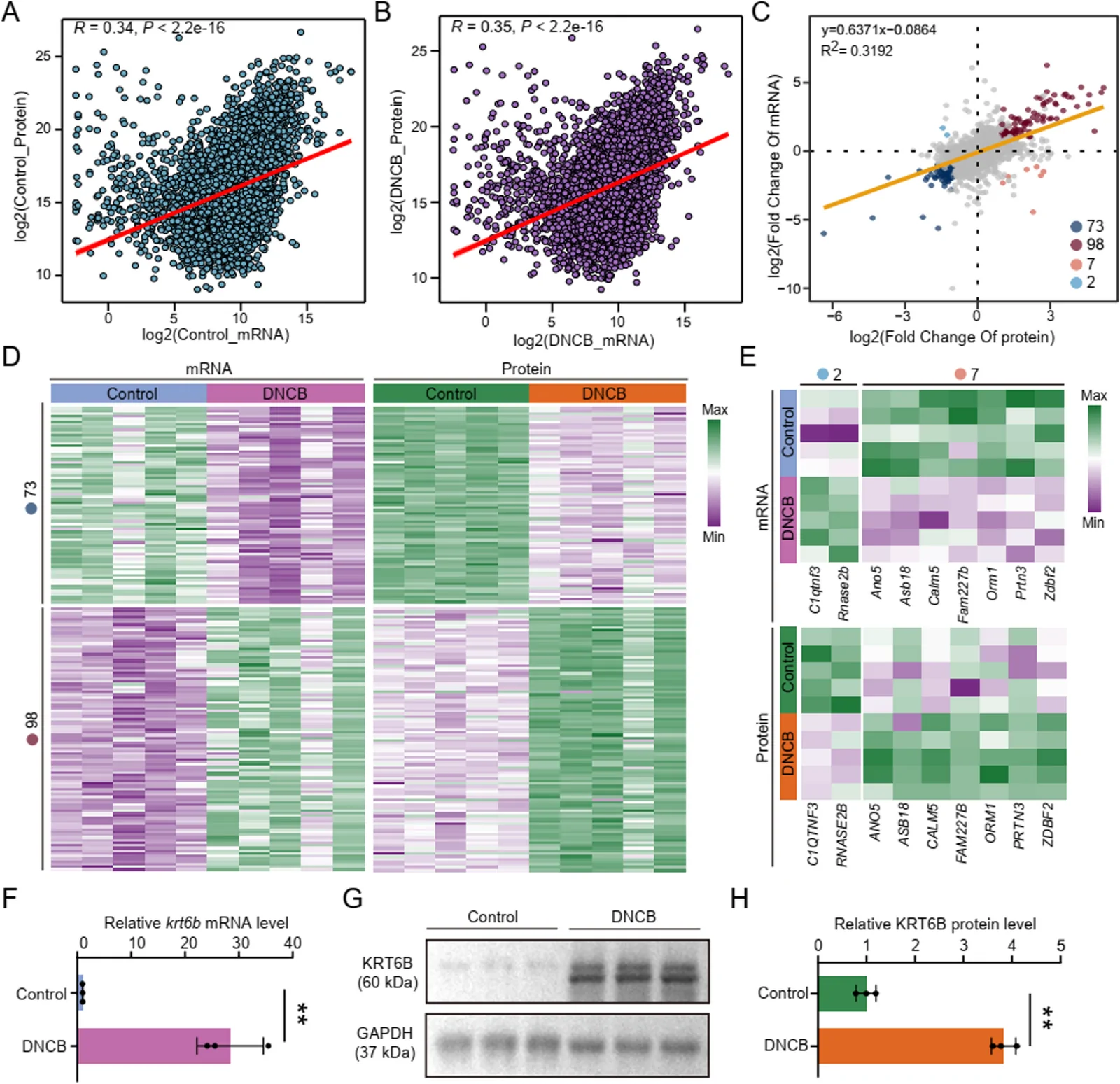
Integration of transcriptomic and proteomic analyses reveals coordinated molecular changes in DNCB-induced skin. **A** Correlation analysis between transcriptomic and proteomic data in the control group. **B** Correlation analysis between transcriptomic and proteomic data in the DNCB-treated group. **C** Scatter plot correlating transcriptomic and proteomic, highlighting molecules with concordant upregulation or downregulation. **D** Heatmap visualizing the expression and abundance levels of molecules with consistent regulation between transcriptomic and proteomic data across control and DNCB-treated groups. **E** Heatmap visualizing the expression and abundance levels of molecules with discordant regulation between transcriptomic and proteomic data across control and DNCB-treated groups. **F** RT-qPCR validation of *Krt6b* expression in control and DNCB-treated skin tissues. **G** Representative Western blot images showing KRT6B and GAPDH protein expression in control and DNCB-treated skin tissues. **H** Densitometric quantification of KRT6B protein abundance normalized to GAPDH.

To assess the concordance between transcriptional and translational responses, we performed correlation analyses of transcriptomic and proteomic data from control and DNCB-treated groups. The correlation coefficients (R) between the two omics layers were highly similar in both the control and DNCB-treated conditions (Fig. 4A and B), indicating a tight coupling of mRNA expression and protein abundance. Integrative analysis of further demonstrated a high degree of consistency between the transcriptomic and proteomic alterations induced by DNCB. Specifically, integration of transcriptomic and proteomic data identified 98 molecules with concordant increases and 73 with concordant decreases (Fig. 4C). Heatmap visualization clearly delineated the distinct molecular profiles between the two groups, with the vast majority of overlapping molecules showing pronounced and coordinated increases or decreases at both expression and abundance levels (Fig. 4D). Only nine molecules exhibited discordant regulation between the two omics layers (Fig. 4E). These findings indicate that DNCB exposure drives a highly concordant reprogramming of both the transcriptome and proteome in skin tissue. To further validate the concordance observed between transcriptomic and proteomic datasets, KRT6B, one of the key molecules identified by the integrative analysis, was selected for orthogonal validation. Consistent with both the RNA-seq and proteomic results, RT-qPCR analysis confirmed significant upregulation of KRT6B mRNA expression in DNCB-treated skin compared with controls (Fig. 4F). Western blot analysis similarly demonstrated increased KRT6B protein abundance in the DNCB group (Fig. 4G and H).

### Functional enrichment and pathway analysis of concordantly altered molecules

**Fig. 5:**
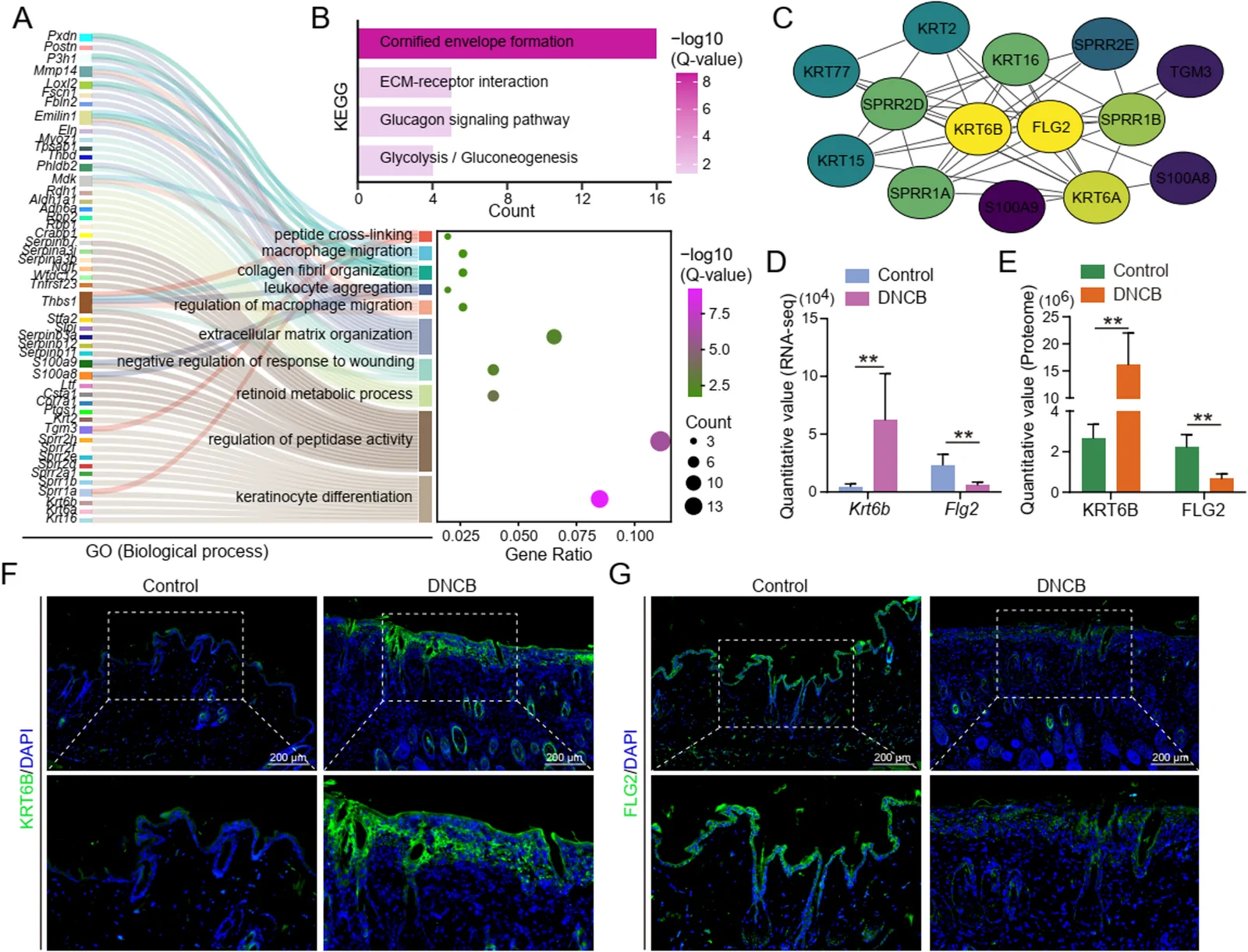
Functional enrichment and validation of core molecules identified through integrative multi-omics analysis.**A** GO biological process enrichment analysis of molecules consistently altered at both transcript and protein levels. **B** KEGG pathway enrichment analysis of molecules consistently altered at both transcript and protein levels. **C** Protein-protein interaction (PPI) network analysis of proteins within the cornified envelope formation pathway. **D** Transcript-level expression validation of the hub genes *Krt6b* and *Flg2* in control and DNCB-treated skin. **E** Protein-level abundance validation of the hub proteins KRT6B and FLG2 in control and DNCB-treated skin. **F** Representative immunofluorescence staining images of KRT6B (green) in the epidermal layers of control and DNCB-treated skin. Nuclei are counterstained with DAPI (blue). Scale bars, 200 μm. **G** Representative immunofluorescence staining images of FLG2 (green) in the epidermal layers of control and DNCB-treated skin. Nuclei are counterstained with DAPI (blue). Scale bars, 200 μm. **: *P* < 0.01.

GO enrichment analysis of the molecules consistently altered at both transcript and protein levels revealed significant enrichment in biological processes critical for epidermal homeostasis, including keratinocyte differentiation and regulation of peptidase activity (Fig. 5A). Subsequent KEGG pathway analysis further indicated that these coordinated molecular changes were prominently enriched in pathways related to cornified envelope formation and ECM-receptor interaction (Fig. 5B).

To delineate the core regulatory network within the significantly enriched cornified envelope formation pathway, a PPI analysis was performed. This identified FLG2 and KRT6B as the top-ranking hub proteins within the network (Fig. 5C). The KRT6B expression was significantly upregulated, whereas FLG2 was markedly downregulated in DNCB-treated skin compared to controls (Fig. 5D and E). This opposing regulation of key structural proteins was further corroborated by immunofluorescence staining, which visually demonstrated the increased KRT6B signal and concomitant decrease in FLG2 in the epidermal layers of DNCB-exposed tissue (Fig. 5F and G).

### Metabolomic profiling reveals altered metabolic pathways in DNCB-induced skin

**Fig. 6:**
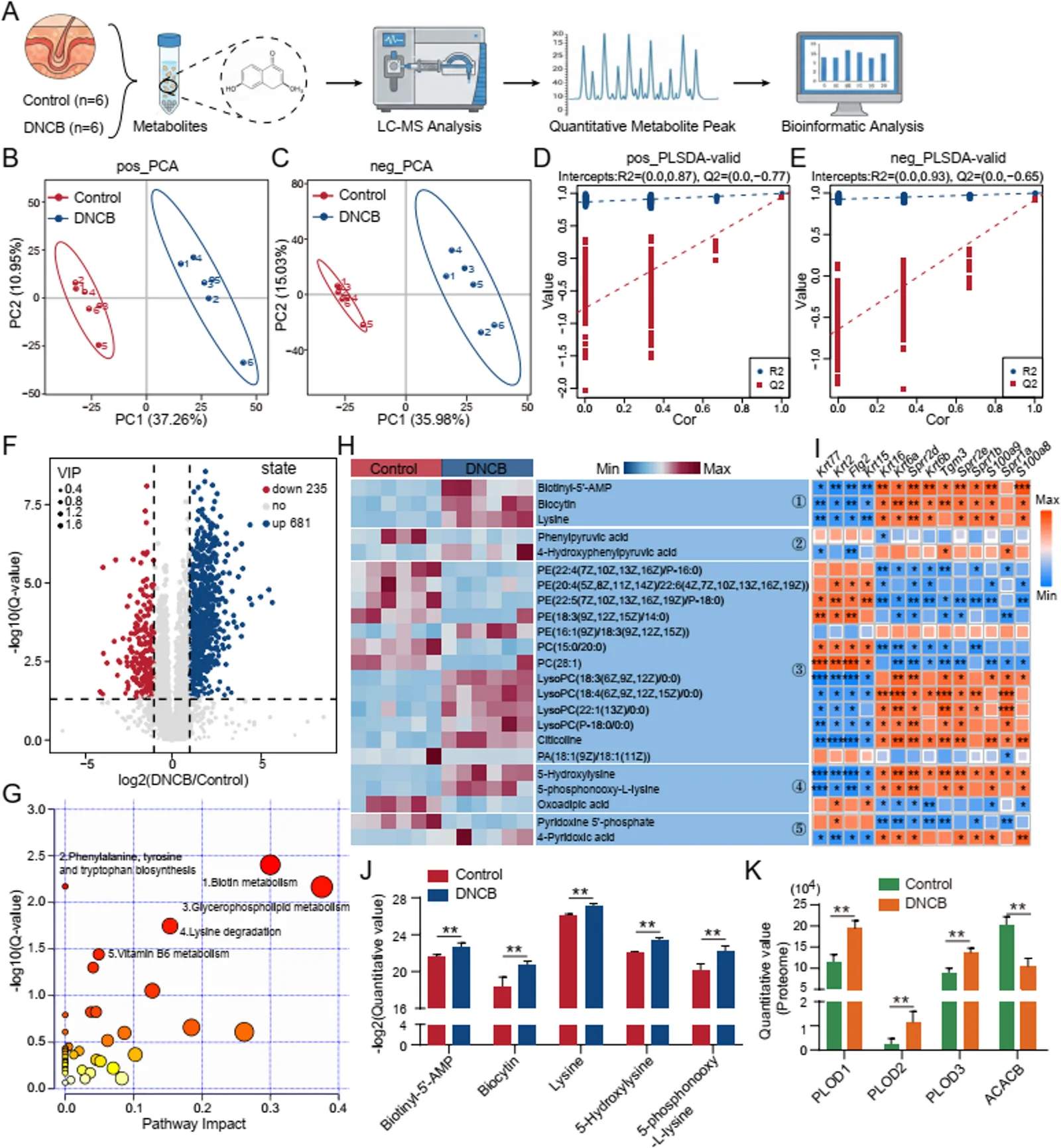
Metabolomic profiling reveals altered metabolic pathways and their association with molecular changes in DNCB-induced skin.**A** Schematic workflow of the comprehensive metabolomic analysis performed on control and DNCB-treated skin tissues. **B-C** The PCA plots of metabolite profiles in positive ionization mode and negative ionization mode, showing separation between control and DNCB-treated groups. **D-E** Partial least squares-discriminant analysis (PLS-DA) score plots in positive ionization mode and negative ionization mode, further demonstrating distinct metabolic clustering between groups. **F** Volcano plot displaying differentially abundant metabolites. **G** KEGG pathway enrichment analysis of the differentially abundant metabolites. **H** Heatmap of the differential metabolites belonging to the enriched KEGG pathways, showing their alteration patterns across control and DNCB-treated groups. **I** Correlation analysis between the levels of differential metabolites and the expression levels of genes involved in the cornified envelope formation pathway. **J** Bar charts showing the relative abundance levels, presented as -log₂(Quantitative value), of selected metabolites (Biotinyl-5’-AMP, Biocytin, Lysine, 5-Hydroxylysine, and 5-phosphonooxy-L-lysine) in control and DNCB-treated groups. **K** Bar charts showing the quantitative protein levels of PLOD1, PLOD2, PLOD3, and ACACB, as determined by proteomic analysis, in control and DNCB-treated skin. **: *P* < 0.01.

Given the established association between AD and lipid metabolism, we performed a comprehensive metabolomic analysis of control and DNCB-treated skin tissues (Fig. 6A). PCA demonstrated a clear separation between the two groups based on overall metabolite profiles in both positive and negative ionization modes (Fig. 6B and C). This distinct metabolic clustering was further corroborated by PLS-DA (Fig. 6D and E).

Volcano plot analysis identified 691 metabolites significantly upregulated and 235 significantly downregulated in DNCB-treated skin compared to controls (Fig. 6F). KEGG pathway enrichment analysis of the differentially abundant metabolites highlighted significant perturbations in several key metabolic pathways, including biotin metabolism, phenylalanine, tyrosine and tryptophan biosynthesis, glycerophospholipid metabolism, lysine degradation, and vitamin B6 metabolism (Fig. 6G).

A heatmap of the differential metabolites belonging to the enriched pathways distinctly illustrated their consistent alteration patterns between the two experimental groups (Fig. 6H). Notably, a significant correlation was observed between these metabolic alterations and the expression levels of genes involved in the cornified envelope formation pathway (Fig. 6I). Of particular interest, specific metabolites including Biotinyl-5’-AMP, Biocytin, Lysine, 5-Hydroxylysine, and 5-phosphonooxy-L-lysine were markedly elevated in the DNCB group (Fig. 6J). These changes were associated with increased expression of PLOD1, PLOD2, and PLOD3 proteins and a concurrent decrease in ACACB protein levels (Fig. 6K), suggesting a coordinated metabolic and proteomic response to DNCB exposure.

## Discussion

This study systematically delineates the complex molecular landscape of skin inflammation in the DNCB-induced AD mouse model through an integrated multi-omics approach. Our data not only validate the fidelity of this model in recapitulating core pathological features of human AD, including epidermal hyperplasia, barrier dysfunction, and immune cell infiltration, but more importantly, uncover a highly coordinated, multi-layered regulatory network through the integration of transcriptomic, proteomic, and metabolomic analyses. This network intricately links structural epidermal disruption, immune activation, and metabolic reprogramming that may collectively driving disease pathogenesis, although causal relationships remain to be established.

The strong concordance between transcriptomic and proteomic alterations, particularly within pathways governing epidermal differentiation, underscores a tightly coupled transcriptional and translational response to DNCB challenge. The most striking and consistent finding across all omics layers was the profound dysregulation of the cornified envelope formation pathway. Key structural components, notably the downregulation of FLG2 and the upregulation of KRT6B, were identified as central hubs in the PPI network. While our study did not include direct functional perturbation of these targets, a substantial body of evidence from independent studies establishes their causal roles in epidermal barrier biology. FLG2 encodes a critical component of the cornified envelope, and its loss-of-function mutations are established genetic risk factors for AD, directly linking FLG2 deficiency to barrier dysfunction [18–20]. Beyond genetic evidence, exogenous recombinant FLG2 has been shown to restore tight junction protein expression, promote keratinocyte differentiation, and attenuate oxidative stress and inflammation in both in vitro and in vivo models, reinforcing its essential role in maintaining skin barrier integrity [21, 22]. In contrast, KRT6B is a well-established marker of keratinocyte activation and hyperproliferation, consistently induced in AD, psoriasis, and wound healing [23–25]. Functional studies have demonstrated that KRT6B-deficient keratinocytes exhibit impaired collective migration and reduced mechanical resilience, indicating a direct role in epidermal repair and barrier maintenance [26]. The consistent enrichment of cornified envelope-related processes across transcriptomic and proteomic supports the presence of impaired epidermal maturation in DNCB-treated skin. Importantly, this molecular pattern is highly consistent with the differentiation defects and barrier abnormalities reported in human AD, reinforcing the translational relevance of the DNCB model [27, 28].

The significant infiltration of CD3⁺ T lymphocytes, F4/80⁺ macrophages, and mast cells confirms a robust immune response. These histological findings are consistent with the immunopathology of human AD, where lesional skin is characterized by infiltration of activated T cells, mast cells, dendritic cells, and macrophages [5, 29]. Notably, the elevated expression of IL33 and IL1RL1 (ST2) observed in our dataset is particularly relevant, as the IL-33/ST2 axis is increasingly recognized as a central driver of type-2 inflammation in AD [30]. Similarly, the increased expression of CCL17, CCL22, and CCR4 is consistent with enhanced recruitment of Th2-polarized lymphocytes, a hallmark feature of AD pathogenesis [31]. Beyond immune activation, both transcriptomic and proteomic analyses revealed substantial extracellular matrix remodeling and protease-related changes. Extracellular matrix remodeling has been recognized as a key regulator of leukocyte trafficking and inflammatory amplification in skin disease [32], whereas dysregulated protease activity contributes to barrier disruption and cytokine activation in AD [18]. This suggests that DNCB exposure does not merely recruit immune cells into a passive tissue but actively reshapes the dermal and junctional architecture, creating a pro-inflammatory microenvironment [33]. The dysregulated ECM may facilitate immune cell migration and retention, while activated complement components can directly amplify inflammation and itch sensation, establishing a self-sustaining cycle [34].

The metabolomic data provide a critical functional layer, revealing significant perturbations in pathways central to cellular metabolism and biosynthesis [35]. The observed alterations in glycerophospholipid metabolism and vitamin/biotin metabolism are of particular relevance [17, 36]. Glycerophospholipids are fundamental to stratum corneum lipid bilayers, and their disturbance has been associated with impaired barrier function in AD [37]. Whether the alterations observed in this study directly compromise barrier integrity in the DNCB model remains to be experimentally tested. Furthermore, the dysregulation of AMPK and PPAR signaling pathways, as evidenced by transcriptomics, connects these metabolic shifts to broader cellular energy sensing and lipid homeostasis [38, 39]. AMPK is a master regulator of cellular energy balance, and its inhibition can promote inflammation and impair differentiation [40]. PPARs are nuclear receptors vital for lipid metabolism and epidermal homeostasis [41]. Their dysregulation in our model aligns with the observed lipid metabolic alterations and impaired barrier function, although a direct causal link has not been established in this study. Notably, the accumulation of specific metabolites like biotin derivatives and lysine pathway intermediates (e.g., 5-Hydroxylysine) correlates with the upregulation of PLOD1-3 enzymes, which are involved in collagen cross-linking and may contribute to ECM stiffness and fibrosis in chronic lesions [32, 42]. Concurrent downregulation of ACACB, the gene encoding acetyl-CoA carboxylase beta, suggests a shift away from mitochondrial oxidative metabolism, a feature increasingly recognized in inflammatory diseases [43, 44]. Targeted pharmacological or genetic perturbation of these metabolic pathways will be an important next step to determine their functional contribution to barrier impairment and inflammation in the DNCB model.

Our integrative multi-omics analysis supports a feed-forward pathogenic circuit in DNCB-induced dermatitis. Chemical hapten-driven immune activation, characterized by Th2-skewed cytokine responses, is associated with perturbation of key metabolic regulators including AMPK and PPAR signaling pathways [14, 45, 46]. Recent functional studies have provided substantial evidence supporting the involvement of these pathways in skin barrier homeostasis and inflammatory responses. Pharmacological activation of PPARα has been shown to normalize epidermal lipid composition, increase filaggrin expression, and improve barrier function in both normal and filaggrin-deficient reconstructed skin models [47]. Consistent with these findings, PPARα activation alleviates inflammation and improves barrier abnormalities in experimental AD models, underscoring its role in epidermal differentiation, lipid metabolism, and cutaneous immune regulation [48, 49]. Similarly, AMPK activation has been reported to suppress allergic skin inflammation through autophagy-related pathways and to enhance epidermal barrier protection in allergic dermatitis models [9, 50]. Given the established roles of these pathways in epidermal lipid synthesis and keratinocyte differentiation [51], their dysregulation may contribute to impaired cornified envelope formation and reduced expression of structural proteins such as FLG2 [52]. Compromised barrier integrity has been associated with increased allergen penetration and cytokine exposure, which may in turn promotes stress keratin expression (e.g., KRT6B) and extracellular matrix remodeling through enzymes such as PLODs [28, 53]. The enrichment of the cytokine-cytokine receptor interaction pathway at the transcriptomic level is consistent with amplification of this inflammatory-metabolic feedback loop. Together with our metabolomic findings, these results support a potential link between metabolic dysregulation, impaired barrier function, and inflammatory responses in DNCB-induced dermatitis, although causal relationships remain to be established.

This study has limitations. The model reflects a chemically induced, acute-to-chronic dermatitis, which may not capture all nuances of the complex, multifactorial etiology of human AD, including the role of specific genetic predispositions like filaggrin (*FLG*) null mutations. Bulk tissue omics averages signals across multiple cell populations and therefore cannot resolve cell-type-specific molecular alterations. The current integration strategy adopted in this study was primarily based on pathway concordance and correlation analyses. While this approach enabled identification of reproducibly altered biological programs across omics layers, it does not resolve causal regulatory relationships or fully exploit the predictive potential of modern multi-omics integration frameworks, such as AI-driven multi-modal integration [54], which represents a future direction for building on the dataset provided here. Accordingly, the present study should be viewed as a foundational molecular resource that defines the multi-layer landscape of DNCB-induced dermatitis and identifies candidate pathways and molecular modules for future investigation. Future integration of single-cell transcriptomics with spatially resolved omics technologies may provide deeper insight into cell-type-specific regulatory mechanisms and further enhance the translational value of these findings. Translating these descriptive multi-omics signatures into clinically actionable biomarkers remains an important long-term goal, and the coordinated molecular alterations identified here may provide a framework for future studies aimed at disease stratification or therapeutic response prediction in AD [13]. Finally, as a hapten-driven model, DNCB may induce pathway-specific molecular responses that do not fully recapitulate all aspects of human AD. Consequently, pathways identified in this study, including AMPK and PPAR signaling, should be interpreted within the context of the experimental model and require further validation in human tissues and mechanistic studies.

## Conclusions

In summary, our integrative multi-omics analysis demonstrates that DNCB-induced dermatitis is characterized by tightly coordinated epidermal structural disruption, immune cell infiltration, and metabolic reprogramming. The convergence of transcriptomic, proteomic, and metabolomic alterations on cornified envelope formation, ECM remodeling, and AMPK/PPAR-associated metabolic pathways underscores the central role of immune metabolic dysregulation in AD pathogenesis. These findings provide a comprehensive molecular framework for understanding barrier dysfunction-driven inflammation and may inform the development of targeted therapeutic strategies for AD.

## Data Availability

Data supporting the findings of this study are available from the corresponding author upon reasonable request.
